# Development and reliability assessment of a new quality appraisal tool for cross-sectional studies using biomarker data (BIOCROSS)

**DOI:** 10.1186/s12874-018-0583-x

**Published:** 2018-11-06

**Authors:** Jan Wirsching, Sophie Graßmann, Fabian Eichelmann, Laura Malin Harms, Matthew Schenk, Eva Barth, Alide Berndzen, Moses Olalekan, Leen Sarmini, Hedwig Zuberer, Krasimira Aleksandrova

**Affiliations:** 1 Nutrition, Immunity and Metabolism Senior Scientist Group, Department of Nutrition and Gerontology, German Institute of Human Nutrition Potsdam-Rehbruecke (DIfE), Arthur-Scheunert-Allee 114-116, 14558 Nuthetal, Germany; 2 University of Potsdam, Institute of Nutritional Science, Potsdam, Germany

**Keywords:** BIOCROSS, Quality appraisal, Evaluation tool, Cross-sectional studies

## Abstract

**Background:**

Biomarker-based analyses are commonly reported in observational epidemiological studies; however currently there are no specific study quality assessment tools to assist evaluation of conducted research. Accounting for study design and biomarker measurement would be important for deriving valid conclusions when conducting systematic data evaluation.

**Methods:**

We developed a study quality assessment tool designed specifically to assess biomarker-based cross-sectional studies (BIOCROSS) and evaluated its inter-rater reliability. The tool includes 10-items covering 5 domains: ‘Study rational’, ‘Design/Methods’, ‘Data analysis’, ‘Data interpretation’ and ‘Biomarker measurement’, aiming to assess different quality features of biomarker cross-sectional studies. To evaluate the inter-rater reliability, 30 studies were distributed among 5 raters and intraclass correlation coefficients (ICC-s) were derived from respective ratings.

**Results:**

The estimated overall ICC between the 5 raters was 0.57 (95% Confidence Interval (CI): 0.38–0.74) indicating a good inter-rater reliability. The ICC-s ranged from 0.11 (95% CI: 0.01–0.27) for the domain ‘Study rational’ to 0.56 (95% CI: 0.40–0.72) for the domain ‘Data interpretation’.

**Conclusion:**

BIOCROSS is a new study quality assessment tool suitable for evaluation of reporting quality from cross-sectional epidemiological studies employing biomarker data. The tool proved to be reliable for use by biomedical scientists with diverse backgrounds and could facilitate comprehensive review of biomarker studies in human research.

**Electronic supplementary material:**

The online version of this article (10.1186/s12874-018-0583-x) contains supplementary material, which is available to authorized users.

## Background

The booming field of biomedical research over the last decades resulted in an increasing number of research papers reporting biomarker information [1, 2]. Biomarkers have been broadly defined as any measurable characteristic of an organism that reflects a particular physiological state. These are molecules isolated from serum, urine, or other fluids that can have multifaceted application (i) indicating the presence or severity of a particular disease state; (ii) evaluation of a therapeutic response and (iii) monitoring disease development. Biomarkers hold great promise for personalized medicine as information gained from diagnostic or prognostic markers can be used to tailor treatment to the individual for highly efficient interventions.

While much of the new molecule discoveries are generated in experimental and laboratory research, epidemiological studies greatly contribute to exploring relevance of identified biomarkers in humans [3–6].

Among different study designs in epidemiology, cross-sectional studies have gained much application in utilizing biomarker data due to their high feasibility. Such studies are relatively easy, fast and cheap to conduct and can provide helpful information for hypothesis generation. In cross-sectional studies both exposures and outcome are measured simultaneously and therefore it may be difficult to determine whether the exposure proceeded or succeeded the outcome. Even though no inferences on causality can be drawn, cross-sectional studies have proven helpful in gaining insights into potential correlations between biomarkers and other factors [5].

Given the abundance of information created through published studies, systematic reviews are often used to summarize and conclusively present obtained knowledge also on biomarkers. Guidelines like the STrengthening the Reporting of OBservational studies in Epidemiology (STROBE) [7] or STROBE-ME [8] have been developed to guide researchers regarding criteria that may help in the conduct of their own research and when evaluating the research of others [7]. However, such guidelines are not suited for obtaining a more objective rating of the study quality. In particular, there is no specific tool to rapidly evaluate study quality in commonly used cross-sectional studies reporting biomarker data.

We recently published a study wherein we report data from a meta-analysis aimed to evaluate correlations between biomarkers [9]. During the analysis, we noticed that many of the peer-reviewed publications that describe uses of biomarkers employ inconsistent methods of analysis and data interpretation, and insufficient reporting on general study procedures (e.g. sample handling, participant selection). However, no tool was suited to assist us in assessing quality of individual studies in all relevant domains. We therefore developed a study quality and reporting assessment tool for biomarker-based cross-sectional studies and evaluated its inter-rater reliability.

## Methods

### Development of BIOCROSS

**Fig. 1:**
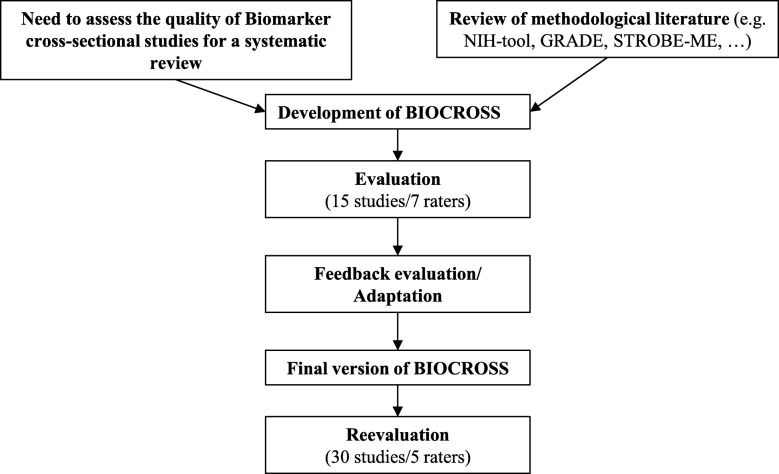
Tool development process of BIOCROSS

The development of BIOCROSS followed a 4-stage approach: 1) design and development, 2) pilot reliability assessment, 3) improvement/adaptation, 4) reliability assessment of the adapted tool (Fig. 1).

As an initial step, a 10-item tool with a crude rating (“1” = positive and “0” = negative) was developed within the work for a systematic review [9]. This tool was partly based on a scale from the National Institutes of Health [10]. Points from the original 14-point evaluation tool were adapted and combined to 7 questions assessing the cross-sectional study design. Furthermore, 3 questions were added assessing biomarker related quality features, namely: ‘Specimen characteristics and assay methods’, ‘Laboratory measurements’ and ‘Biomarker data modeling’.

As a second step, pilot reliability was conducted among 7 raters assessing 15 studies to measure the inter-rater reliability. Written explanations were provided to explain each of the item-related questions. The raters were asked to give feedback on the applicability of the tool within 2 weeks.

Eligible raters were supposed to have a scientific background (at least bachelor's degree in an epidemiological, biomedical or nutritional area)*.* Furthermore, a briefing of how to use BIOCROSS was provided. The studies were chosen randomly from a pool of 77 studies used for a systematic review conducted within our working group [9].

As a third step, we improved and adapted the tool following results from the pilot inter-rater reliability testing. Raters’ feedbacks were analyzed, and the tool was revised leading to the final version of BIOCROSS.

The major changes that were implemented included changing the dichotomous evaluation scale (“0” or “1” points) towards an ordinal scale (“0”, “1” or “2” points) leading to a maximum of 20 points. This was done to allow a more gradual rating of studies to avoid unjustified low scores as all items needed to be covered to receive a point. Three ‘issues to consider’ (IC) are provided for each of the 10 items. If all IC were discussed in a feasible way, a score of “2” should be awarded, if 1 or 2 IC were discussed, 1 point otherwise 0 points should be awarded. By allowing a gradual rating, the precision of our tool should be improved. Additional textual edits were also made to improve general understanding of each item.

As a final step, the adapted tool was handed out to 5 raters to assess 30 studies over a period of 4 weeks. These studies were chosen semi-randomly from the same pool as the first evaluation. Results from the first evaluation were used to assure, that studies of different levels of reporting quality are being assessed.

Briefing was not provided as it did not seem to have added additional value at the first reliability assessment. We therefore assessed the use of the tool among raters deploying the information provided in the written “User’s guide to BIOCROSS” without preliminary extensive training (see Additional file 1).

### Statistical analysis

To assess inter-rater agreement, a two-way intraclass correlation coefficient (ICC) was used to calculate the agreement among raters for the total BIOCROSS score as well as for each of the evaluated domains. The inter-rater-reliability (IRR), as proposed by Cicchetti et al. [11], provides cutoff points for qualitative ratings of agreement based on ICC values. An IRR is treated as excellent for values between 1.0 and 0.75, good for values between 0.74 and 0.60, fair for values between 0.59 and 0.40, and poor for values below 0.40. All statistical analyses were performed using computing environment R (R Development Core Team, 2013) packages “irr” [12] and “lpSolve” [13].

## Results

### BIOCROSS evaluation tool

The tool has been divided into 5 domains (‘Study rational’,’ Design/Methods’, ‘Data analysis’, ‘Data interpretation’ and ‘Biomarker measurement’), aiming to assess different quality features of biomarker cross-sectional studies. Detailed explanations on how to score different items have been provided (see Additional file 1).

### Domains description

The first domain, ‘Study rational’ deals with evaluation of the study objective and pre-specified research hypotheses. It looks into how the introduction provides important information in order to get an idea of what to expect from the study.

The second domain, ‘Design/Methods’ consists of two items assessing study population selection and representativeness. The first item assesses how the study population selection was performed and how information about the selection process is presented in the publication. The second item of this domain addresses the representativeness of the study population, evaluating sampling frame, participation rate as well as the sample size justification which should be provided by the authors.

The third domain, ‘Data analysis’ consists of two items aimed to assess how data analysis was performed. The first item assesses the description of study population characteristics. It assesses if important information about the study population is presented in a feasible way, if exposures and potential confounders are named and described and if values were excluded and what strategies were applied to address that issue. The second item assesses evaluation of the pertaining methods for statistical analysis.

The forth domain, ‘data interpretation’ consists of two items assessing the interpretation and evaluation of the results as well as potential study limitations. The first item addresses the issue of interpretation of the results in the context of pre-specified research hypotheses. The item assesses if results are interpreted in the context of similar studies (if such studies exist) and how the biological context of the biomarkers under investigation is described. The second item addresses the issue of how study limitation arising from the cross-sectional study design and the need of consistency with similar findings were discussed.

The fifth domain, ‘Biomarker measurement’ consists of three items that assess how measurement, handling and modelling of biomarkers were performed. The first item addresses the specimen characteristics, handling and assay methods used to perform analysis. It assesses if a reproducibility assessment was performed to evaluate biomarker stability and the quantitation methods used in the analysis. The second item assesses the laboratory measurement itself. The questions addressed are whether the place of measurement, the quality control procedures or the coefficient of variation of biomarker measurements have been provided in the paper. The third item assesses adequacy of statistical analyses, outlier handling as well as possible errors resulting from biomarker measurement inaccuracies and how these have been discussed in the publication (Table 1).

**Table 1:** BIOCROSS evaluation tool. Depicted is the BIOCROSS evaluation tool aimed at evaluating the quality of reporting of biomarker cross sectional studies

Item	Issues to consider (IC)	Study quality feature
	1st Domain: Study rational	
1.	1.1 Was the biomarker under study described? 1.2 Was the rationale for the study (research question) clearly presented? 1.3 Were the study objectives/ hypothesis clearly stated?	Hypothesis/Objective
	2nd Domain: Design/Methods	
2.	2.1 Were the characteristics of the study participants presented? 2.2 Were the disease stages or comorbidities of the included participants described? 2.3 Were the inclusion and exclusion criteria for study participation defined?	Study population selection
3.	3.1 Was the sampling frame reported (study population source) 3.2 Was the participation rate reported (i.e. eligible persons at least 50%)? 3.3 Was sample size justification or power description provided?	Study population representativeness
	3rd Domain: Data analysis	
4.	4.1 Were the study population characteristics (i.e. demographic, clinical and social) presented? 4.2 Were the exposures and potential confounders described? 4.3 Were any missing values and strategies to deal with missing data reported?	Study population characteristics
5.	5.1 Did the authors clearly report statistical methods used to calculate estimates (e.g. Spearman/Pearson/Linear regression, etc.)? 5.2 Were key potential confounding variables measured and adjusted statistically in reported analyses? 5.3 Was the raw effect size estimate (correlation coefficient, beta coefficient) or measure of study precision provided (e.g. confidence intervals, precise (!) *p*-value*)?	Statistical analysis
	4th Domain: Data interpretation	
6.	6.1 Was the data discussed in the context of study objectives/hypotheses? 6.2 Was the interpretation of the results considering findings from similar studies? 6.3 Was the biological context described?	Interpretation and evaluation of results
7.	7.1 Was the cross-sectional nature of the analysis discussed? 7.2 Did the authors acknowledge restricted interpretation due to measurements at one point in time and no statement about causality possible using cross-sectional studies? 7.3 Did the authors acknowledge need for consistency with other research?	Study limitations
	5th Domain: Biomarker measurement	
8.	8.1 Were the measurement methods described? (assay methods, preservation and storage, detailed protocol, including specific reagents or kits used) 8.2 Were the reproducibility assessments performed for evaluating biomarker stability? 8.3 Were the quantitation methods well described?	Specimen characteristics and assay methods
9.	9.1 Was the laboratory/place of measurement mentioned? 9.2 Were any quality control procedures and results reported (e.g. reported coefficient of variation? 9.3 Were the analyses blinded for laboratory staff?	Laboratory measurement
10.	10.1 Was the distribution of biomarker data reported (if non-normal how it was standardized)? 10.2 Did the authors report on methods or outlier detection and handling? 10.3 Were any possible errors resulting from measurement inaccuracies discussed?	Biomarker data modeling

*Reporting not significant (ns) or *p* > 0.05 is not precise and does not allow a judgment on precision

### Agreement and reliability

Total scores of BIOCROSS for each study used in the evaluation process are depicted in Table 2. The average values were between 8.2 for study 24 and 15.8 for study 29. Maximum values ranged from 10 (study 5) to 19 (study 29). Minimum values ranged from 7 (study 5) to 13 (study 21). The smallest difference between different raters was 2 points (6 studies) and the biggest difference was 7 points (study 29). Standard deviations ranged from 0.69 (Study 6) to 2.11 (Study 29). The overall mean was 12.29.

**Table 2:** Total scores for each evaluated study

Study^a^	Ratings				
	Mean	SD	Min	Max	Median
1	11.3	0.75	10	12	11.5
2	12.3	1.70	9	14	12.5
3	14.0	1.29	12	16	14
4	12.3	1.70	11	16	12
5	8.7	0.94	7	10	9
6	10.8	0.69	10	12	11
7	12.8	1.34	10	14	13
8	12.8	1.07	11	14	13
9	11.8	0.90	10	13	12
10	12.3	0.94	11	14	12
11	13.8	1.46	11	15	14.5
12	13.8	1.07	12	15	14
13	13.8	1.07	12	15	14
14	12.5	0.76	11	13	13
15	9.3	0.75	8	10	9.5
16	11.8	1.46	9	13	12.5
17	9.2	0.90	8	11	9
18	14.5	1.89	11	17	14.5
19	14.0	1.15	13	16	13.5
20	14.7	1.80	12	17	15
21	15.7	2.05	13	18	15.5
22	11.2	0.90	10	12	11.5
23	9.8	1.07	8	11	10
24	8.2	1.07	7	10	8
25	11.8	2.03	9	15	12
26	13.3	0.94	12	14	14
27	10.5	1.26	9	13	10
28	13.7	1.37	12	16	13
29	15.8	2.11	12	19	16
30	11.8	1.57	9	14	12

^a^Studies used in the evaluation process of BIOCROSS

Table 2: Total scores for each study used in the evaluation process. Mean: mean of all raters, SD: Standard deviation, Min: minimum rating, Max: maximum rating, Median: median of all raters.

**Fig. 2:**
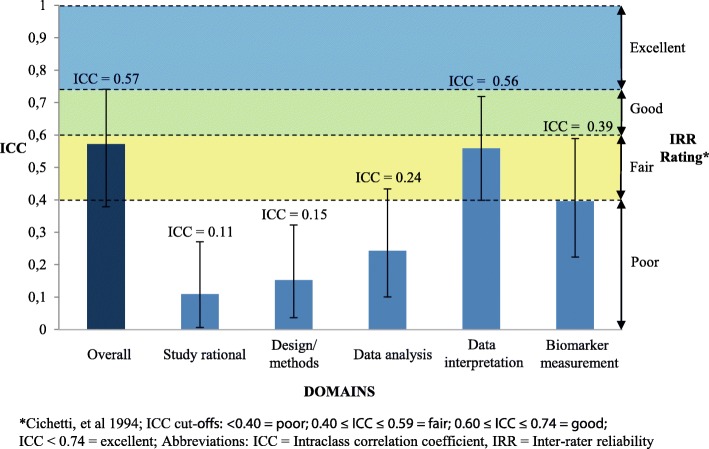
ICC Scores (95% CI) and Inter-Rater reliability ratings of BIOCROSS

The ICCs representing agreement among raters for the overall assessment score, as well as the agreements for each of the 5 domains are depicted in Fig. 2. Datasets to reproduce the calculations are provided as a .txt file (see Additional file 2). The inter-rater agreement was good with an estimated ICC of 0.57 (95% CI: 0.38–0.74). Among individual domains, the ICCs varied such that lowest agreement was observed for the three domains, ‘Study rational’: ICC = 0.11 (95% CI: 0.01–0.27), ‘Design/Methods’: ICC = 0.15 (95% CI: 0.04–0.32) and ‘Data analysis’: ICC 0.24 (95% CI: 0.10–0.43). The raters seem to agree most on two of the domains: ‘Data interpretation’: ICC 0.56 (95% CI: 0.40–0.72) and ‘Biomarker measurement’: ICC 0.39 (95% CI: 0.22–0.58). As compared with the pilot test tool, an overall 30% increase of the ICC (0.44 to 0.57) could be seen for the revised tool. Among different domains, the most prominent improvement (124% increase) could be achieved within the domain ‘data interpretation’ (ICCs range: from 0.25 to 0.56). The ICCs for the rest of the domains were not substantially changed. Within the domain Biomarker measurement, the item “Specimen characteristics and assay methods” as well as the item “Biomarker data modeling” could only reach ICCs of 0.12 (95% CI: 0.02–0.28) and 0.23 (95% CI: 0.08–0.42) respectively.

We also examined if items were not discriminatory, meaning that they did not fully utilize the scale of our tool. This could be proven for the items 1, 5 and 6 in which the lowest grading of “0” was not awarded for any of the studies under investigation.

Figure 2 Intraclass correlation coefficient (ICC) scores and inter-rater reliability ratings of BIOCROSS total score and individual domains; BIOCROSS was divided into 5 different domains meant to assess different aspects of a cross-sectional study. ‘Data rationale’ is meant to assess how the authors presented the study objective and how the hypothesis was defined. ‘Design/Methods’ assesses study population selection and representativeness. ‘Data analysis’ investigates how study population characteristics were presented and how statistical analysis was performed. ‘Data interpretation’ deals with evaluation and interpretation of results and the discussion of study limitations, due to the cross-sectional design of the study. ‘Biomarker measurement’ assesses how specific biomarker characteristics and assay methods were presented and how laboratory measurements as well as biomarker data analysis were performed.

The average time needed by the reviewers to complete one evaluation was 13.55 min. There were substantial differences between the raters, with the fastest rater using an average of less than 10 min per study and the slowest rater needing on average of more than 20 min per study. The level of experience and the time needed to complete one evaluation were correlated. As expected, more experienced reviewers were faster in reading and evaluating studies.

The raters have been also asked to provide a feedback on their experiences of using the BIOCROSS tool. The most important point raised by the reviewers was the need for re-formulation of several item explanations in the ‘User’s guide’ to BIOCROSS. These have been updated and further clarified in the revised version of BIOCROSS.

## Discussion

BIOCROSS was developed as a tool designed for use by biomedical specialists to assess the quality and reporting of biomarker-based cross-sectional studies. BIOCROSS combines 10 items within 5 study evaluation domains ranging from study rationale and design to biomarker assessment and data interpretation scoring for a maximum score of 20 points. The tool could be suggested as a reliable and valid method for assessing study reporting quality and its further application could assist researchers in the conduct of systematic reviews and meta-analyses within the rapidly evolving field of biomarker research.

In the past years marked by the establishment of ‘evidence-based medicine’, the assessment of study quality of conducted research has turned into a subject of an intensive effort and exchange of scientific guidelines and recommendations. These recommendations have been largely aimed to assist researchers and policy makers in the decision process on which studies to include into their analysis [14–17].

In a previous review of tools assessing the risk of bias in observational research the lack of an easily applicable tool to assess biomarker-based observational studies has been acknowledged [18]. Based on it, the following recommendations have been issued: [1] to focus on the development of tools with small number of key domains; [2] to address particular study design and topic areas; [3] to use simple checklist [4] to ensure that the tools undergo a careful development phase and [5] to evaluate the developed tools in terms of their validity and reliability. In our approach we largely followed these recommendations and adapted them specifically to assessing quality of reporting of biomarker-based cross-sectional studies.

We are not the first to address the quality of studies in epidemiological research. Previously several tools have been developed, some of which focusing on general aspects of reporting across different disciplines (i.e. Consolidated Standards of Reporting Trials (CONSORT) [19]), while others focusing on specific research topics (i.e. Quality Assessment of Diagnostic Accuracy Studies (QUADAS) [17]). Despite guidelines like STROBE-ME [8] could be helpful in designing future planned biomarker studies, they are not meant for assessing the quality of previously conducted studies. Similar to our approach was used by the Biomonitoring, Environmental Epidemiology, and Short-lived Chemicals tool (BEES-C) aimed at evaluating studies dealing with short-lived chemicals, including biomarkers [20, 21]. It consists of 14 study assessment components which can be scored with a three-point scale (Tier1, Tier2, and Tier3). However, as compared to BIOCROSS, BEES-C mostly focuses on biomarker selection and measurement issues, deploying 8 of 14 points to this section, while BIOCROSS attempts to provide an overall assessment of the study and not particularly focusing on biomarker measurements. Furthermore, BIOCROSS was developed to be easily applicable by professionals without practical training in epidemiology and biostatistics allowing an effective use within a short period of time. BIOCROSS focuses on the evaluation of most commonly reported biomarker association study design in the current flow of biomedical literature: cross-sectional studies; however, it may be also applicable to other observational study designs. Moreover, as compared to previous tools, such as the ‘BEES-C’, BIOCROSS was deliberately validated to allow a critical evaluation of the obtained data.

One potential use of our tool in the future would be to evaluate quality of reporting of biomarker-based epidemiological studies. Evaluation tools, assessing the quality of reporting are necessary if the quality of studies to be included into systematic reviews needs to be reviewed. As systematic analyses are only as good as the studies used to derive the data, proper quality assessment is necessary to assure a high quality of systematic analyses. Such quality assessments should be conducted through validated tools and reported within meta-analyses. A lack of quality appraisal can lead to misleading interpretations of data. This is especially important in strongly developing and changing fields of research, as new scientific knowledge and new experimental technics need to be considered in the evaluation.

The qualitative analysis based on the overall feedback of the raters pointed to a positive experience of tool application. Most of the reviewers stated, that the tool was easy and relatively fast to apply. Below we discuss some of the issues raised by the raters.

Three raters stated, that our rating with awarding 1 point if 1 or 2 IC were discussed, leaded to unjustified high scorings of some studies. A change to: 0 and 1 IC = 0 points, 2 IC = 1 point and 3 IC = 2 points was therefore suggested by the raters. The change in the scoring may contribute to an increased discrimination within several items and may be considered by other researchers. Two raters stated, that even though the tool was easy to conduct, it might be difficult to use, as the quality of reporting does not necessarily provide valid information about the actual quality of the data. Furthermore, some unclear formulations in the “User’s guide to BIOCROSS” were mentioned. These points raised by the raters were discussed and clarified and were taken into account in the revised version of the “User’s guide to BIOCROSS” (Additional file 1).

BIOCROSS has several strengths. The most important is that it combines the evaluation of cross-sectional study design with specific characteristics of biomarker-based studies. BIOCROSS is a freely accessible and ready to use evaluation tool. No extensive training is necessary, as conclusive descriptions for each point are provided and freely accessible. Furthermore, the tool has been validated making it possible to critically evaluate obtained data from scoring papers, e.g. for a systematic review. With an average rating time of around 13 min per study, BIOCROSS is relatively fast to conduct and also suitable to rate a large amount of studies.

There are also several limitations and weaknesses of BIOCROSS. As ratings differed considerably especially within certain domains, personal experience seems to strongly influence the rater’s decision how to grade specific items. As BIOCROSS mostly assesses the quality of reporting, it is difficult to draw conclusions on the actual quality of obtained data. Therefore, the evaluation of obtained data might be subjective, raising questions about how to use this data in a systematic review. We decided against an intensive training of our reviewers which may have contributed to a lower level of agreement for some of the domains. However, in reality organizing training would be laborious, time-consuming and hard to implement. We have been therefore interested to evaluate a more applicable approach such as the use of a ‘User’s guide’. Furthermore, to reflect a real situation we also chose reviewers with different scientific backgrounds and level of education.

As BIOCROSS asks for specific items to be included into the paper, missing some of them can dramatically reduce the obtained scores. To address this problem, a gradual rating was introduced which allows to evaluate the quality more precisely. On the other hand, discussing points considered important could lead to a relatively high score even though important points like statistical evaluation of presentation of the data seem to be poor. This is especially prominent for items 1, 5 and 6 which were not discriminating in our analysis. To address this problem, we suggest that authors use the information provided through the IC to assess a potential risk of bias and then decide if they want to use such a study for their analysis. As any quality assessment tool BIOCROSS will be an organic item that can change if improvement is needed. We understand the problematic of tools evaluating the quality of research articles and are aware that no tool can be perfectly objective.

## Conclusion

BIOCROSS is a new quality appraisal tool suitable for assessment of evidence from cross-sectional epidemiological studies employing biomarker data. The tool is reliable for use by biomedical scientists and could be applied to facilitate comprehensive review of biomarker studies in human research.

## Additional files

Additional file 1: Scoring system of BIOCROSS: A User’s Guide to BIOCROSS. Point-by-point description to enable the application of the BIOCROSS tool. (DOCX 24 kb)

Additional file 2: Dataset and R code. All executed R code and rater results. (TXT 37 kb)

## Abbreviation

BEES-C: Biomonitoring Environmental Epidemiology and Short-lived Chemicals
CI: Confidence interval
CONSORT: Consolidated Standards of Reporting Trials
IC: Issue to consider
ICC: Intraclass correlation coefficients
IRR: Inter-rater-reliability
Max: Maximum rating
ME: Molecular Epidemiology
Min: Minimum rating
NIH: National Institutes of Health
QUADAS: Quality Assessment of Diagnostic Accuracy Studies
SD: Standard deviation
STROBE: STrengthening the Reporting of OBservational studies in Epidemiology—
