# Supplementary material for: Development and reliability assessment of a new quality appraisal tool for cross-sectional studies using biomarker data (BIOCROSS)
Source: BMC Med Res Methodol. 2018 Nov 6;18:122. doi: 10.1186/s12874-018-0583-x (PMC6219097; doi:10.1186/s12874-018-0583-x)
Supplement: Supplementary file 1 — Scoring system of BIOCROSS: A User’s Guide to BIOCROSS. Point-by-point description to enable the application of the BIOCROSS tool. (DOCX 24 kb) [file 12874_2018_583_MOESM1_ESM.docx]

**Additional file 1**

# **Scoring system of BIOCROSS: A User’s Guide to BIOCROSS**

BIOCROSS includes 10 items (I1 – I10) grouped into 5 domains to assess different quality features of reporting of biomarker cross-sectional studies. Items 1-7 deal with quality features which could be applied for all kind of cross-sectional analyses. Here the items are divided into 4 different domains namely “Study rational”, “Design / Methods”, “Data analysis” and “Data interpretation”. Items 8-10 deal specifically with biomarker associated issues grouped into the domain “Biomarker measurement”. Information to rate each item may be found throughout the whole paper. The STROBE (1) as well as the STROBE-ME statement (2), the NIH – Study Quality assessment tools (3) and tools from the grade working group (4) were used as inspiration for our tool.

***Study rational:*** 1) Hypothesis/Objective

***Design / Methods:*** 2) Study population selection 3) Study population representativeness

***Data analysis:*** 4) Study population characteristics 5) Statistical analyses

***Data interpretation:*** 6) Interpretation and evaluation of results 7) Study limitations

***Biomarker measurement:*** 8) Specimen characteristics and assay methods 9) Laboratory measurement 10) Biomarker data modeling.

Three “Issues to consider” (IC) are provided for each item to help assessing this specific study quality feature. The mentioning of all issues should lead to a score of “2”. If one or two issues are missing, a reduced score of “1” should be awarded. Not mentioning any of the issues should lead to a score of “0”. Not all points need to be discussed in detail! The idea is to examine if the authors thought about specific points and how they reported these points in the paper.

***Study rationale.***

1) *Feature:* Hypothesis/Objective

In this item, the authors of the paper should clearly describe the biomarker under investigation. Important characteristics, physiological function or pathophysiological implications may be considered. This is important as the nature of a biomarker and its physiological/pathophysiological role provides important information for researchers who are reading the paper (IC 1.1). It should state the research question and give an explanation why the cross-sectional study was carried out. To get this information straight at the beginning provides the reader with an idea of what to expect from the study and helps in the overall understanding of the paper (IC 1.2). Furthermore, study objectives as well as pre-specified hypotheses should be mentioned in the introduction. This is important, as results should later be discussed upon the objectives and the pre-specified hypotheses stated in the introduction, helping the reader to grasp a clear understanding of what the authors want to communicate. (IC 1.3)

***Design / Methods.***

2) *Feature:* Study population selection

In the second item, the study participants are in the focus. Special characteristics (e.g. children, juvenile/aged people, male/female ratio, etc.) (IC 2.1) and the overall disease stage or possible comorbidities (e.g. healthy population or population with underlying (pathologic) conditions like obesity, cardio vascular diseases or stroke patients, etc.) of the included participants should be described, as these could later confound results if not accounted for in the analysis. These factors should be described in a clearly understandable way (IC 2.2). The authors should also name and apply inclusion and exclusion criteria. This information is important to assess possible population bias within the study population and is helpful to evaluate the results of the study (IC 2.3)

3) *Feature:* Study population representativeness:

In order to evaluate the repetitiveness of the study population, the authors need to report the source they drew their study population (sampling frame) (IC 3.1). From this sampling frame, a participation rate of at least 50% should be achieved (IC 3.2). These points are important to reduce the possibility of selection bias. Furthermore, a power calculation or sample size justification should have been performed and the results should be reported to ensure that statistically significant results can be obtained within the study (IC 3.3). Information should be presented in a feasible way that allows the reader an easy evaluation of these study quality features.

***Data analysis.***

4) *Feature:* Study population characteristics

In the results part the study population should be further characterized. Information about the study population is normally given in a table which summarizes important information (IC 4.1). Exposures and potential confounders should also be named and defined, as they are important for statistical evaluation and interpretation of the results. This information can also be included in the text (IC 4.2). It is important to state if values were missing or excluded (e.g. example due to measurement limits or missing feedback of study participants) and what strategies were applied to deal with this issue. Excluding values without stating reasons makes it difficult for the reader to evaluate possible selection bias resulting from measurement or compliance issues (IC 4.3).

5) *Feature:* Statistical analyses

Statistical analyses should be adequately performed and presented in order to allow the reader to evaluate the results based on broadly accepted statistical approaches. When reporting cross-sectional associations, the authors should have clearly described the type of correlation (e.g. Spearman, Pearson correlation, linear or logistic regression (depending on distribution of the biomarker), generalized linear models to assess multiple category outcomes, receiver operating characteristics (ROC) curve which can be transformed into a Matthews correlation coefficient, etc.) In case biomarker panels were used in the analyses, the authors should report how this correlation coefficients were obtained. To obtain reliable results for multiple biomarkers either independent testing sets or bootstrap resampling/cross-validation procedures should be conducted. These procedures create multiple datasets of the original one using random (re)sampling (5).(IC 5.1). The authors should have measured key potential confounding variables to adjust the obtained correlations. These should be clearly reported in the analysis (IC 5.2). Furthermore, a test on statistical significance should be performed and properly reported. Reporting the raw beta-coefficient before and after adjustment for covariates in models is preferred to reporting formal statistical significance alone (P-value and confidence limits) in order to allow readers own data interpretation. If P-value is used to test for statistical significance, a precise (<0.05) P-value should be reported in order to allow an evaluation of the results. If results show Pvalues above 0.05, this should be properly discussed and explained in the paper and a statement that the results are not significant should be provided. This also counts for measurement issues. Reporting non-significant results and later using these measurements to establish (significant) correlations should be avoided (IC 5.3)

***Data interpretation.***

6) *Feature:* Interpretation and evaluation of results

The obtained results should be discussed in the context of pre-specified hypotheses given in the introduction. Therefore, hypotheses have to be given in the introduction. If these are missing, this requirement cannot be fulfilled (IC 6.1). Furthermore, the results should be put into context with findings from similar studies (if applicable and similar or related studies were carried out before). Such studies should be used to reflect the own results and critically evaluated the own findings (IC 6.2). As biomarkers are the main focus of the study, the biological context should be discussed and considered for interpreting study results. The authors should discuss possible confounding variables, physiological and pathophysiological significance as well as interferences which could arise from these aspects (IC 6.3).

7) *Feature:* Study limitations

The authors should be clear about limitations. They should acknowledge the cross-sectional nature of the analyses as such a study design bears several limitations (IC 7.1). No statements about causality of association can be made in a cross-sectional study. This is due to the measurement at one point in time does not allow temporal analyses. This should be clearly stated by the author and the reporting and interpretation of results should be moderate to avoid implications about causal association (IC 7.2). The author should acknowledge the need for consistency with other research. Furthermore, the representativeness of the study population should be discussed, as each study examines subjects from a certain background (cultural, genetic, social, etc.). This could be problematic if results are applied to other populations differing from this specific study population. It should be discussed how the data obtained can be or have already been replicated in other populations, using other biomarker measurement methods or after accounting for a different set of confounders. A statement about potential replication of study results would show that the authors considered the need to evaluate consistency of their findings (IC 7.3).

***Biomarker measurement.***

8) *Feature:* Specimen characteristics and assay methods

The biological material and assay methods should be clearly described. Here it is important, that the assay method (e.g. HPLC, LCMS, ELISA, Fluorescence dependent methods, Flow cytometry, etc.), the preservation and storage protocols (e.g. storage temperatures, were certain chemicals added to the samples, when and how were the samples processed, etc.) are mentioned. Furthermore, either a detailed protocol including specific reagents or the kits used should be reported. This is important to allow other researcher to evaluate possible sources of error resulting from measurement or preservation/handling of the samples. A reference to further studies might also be used. If this is the case, these studies should be evaluated (IC 8.1). A reproducibility assessment should be performed to evaluate the biomarker stability. In case other researches want to conduct similar studies, it is very important to know beforehand how to handle biomarkers of interest (IC 8.2). Quantitation methods should also be stated, so that the reader knows how measurement values were generated. As different quantitation methods (e.g. photometry, mass spectrometry, etc.) vary in sensitivity and might itself have an impact on the biomarker (e.g. several photometric measurements of light sensitive substances), this is important to evaluate the quality of information (IC 8.3).

9) *Feature:* Laboratory measurements

Laboratory measurements should be clearly described. Therefore, the laboratory/place of measurement should be mentioned. This could help other research groups if they want to conduct similar studies and it might explain contradicting results, which might be due to systematic errors resulting from special conditions in this laboratory setting (IC 9.1). Quality control procedures and results should be tested and reported in a study. Here the coefficient of variation could be given to allow an evaluation of the results (IC 9.2). To improve analyses, the laboratory stuff should be blinded, as some evaluations might be prone to error if certain characteristics of the study participants are known (e.g. micronuclei determination, comet assay, setting borders in fluorescence cytometry, etc.) (IC 9.3).

10) *Feature:* Biomarker Modeling

This item deals with the adequate handling of biomarkers in statistical analyses as well as the presentation of the data. The authors should report how the biomarkers were distributed. If the biomarkers were non-normally distributed, what measures for standardization were deployed (IC 10.1)? The authors should deploy and report methods to detect and handle outliers (e.g. min/max detection limits, cut-off points for inflammatory biomarkers to exclude participants with acute infections, etc.). This is important to avoid results that are confounded by unknown, underlying pathological conditions (IC 10.2). Any possible errors resulting from measurement inaccuracies should be discussed. A statement that deals with possible errors resulting from measurements inaccuracies or variations between different kits should be included in the discussion (IC 10.3).

**References**

1. von Elm E, Altman DG, Egger M, Pocock SJ, Gotzsche PC, Vandenbroucke JP, et al. The Strengthening the Reporting of Observational Studies in Epidemiology (STROBE) statement: guidelines for reporting observational studies. PLoS medicine. 2007 Oct 16;4(10):e296. PubMed PMID: 17941714. Pubmed Central PMCID: 2020495.
2. Gallo V, Egger M, McCormack V, Farmer PB, Ioannidis JP, Kirsch-Volders M, et al.

STrengthening the Reporting of OBservational studies in Epidemiology--Molecular Epidemiology (STROBE-ME): an extension of the STROBE Statement. PLoS medicine. 2011 Oct;8(10):e1001117. PubMed PMID: 22039356. Pubmed Central PMCID: 3201942.

3. National institutes of Health/National Heart LaBI. Quality Assessment Tool for Observational Cohord and Cross-Sectional Studies [updated March 2014; cited 2017 06.11.2017].
4. Guyatt GH, Oxman AD, Vist GE, Kunz R, Falck-Ytter Y, Alonso-Coello P, et al. GRADE: an emerging consensus on rating quality of evidence and strength of recommendations. Bmj. 2008 Apr 26;336(7650):924-6. PubMed PMID: 18436948. Pubmed Central PMCID: 2335261.
5. Bland, J. Martin; Altman, Douglas G. (2015): Statistics Notes. Bootstrap resampling methods. In *BMJ (Clinical research ed.)* 350, h2622. DOI: 10.1136/bmj.h2622.
